# Treating postextubation dysphagia after stroke with pharyngeal electrical stimulation –insights from a randomized controlled pilot trial

**DOI:** 10.1016/j.neurot.2025.e00613

**Published:** 2025-05-17

**Authors:** Sonja Suntrup-Krueger, Bendix Labeit, Jonas von Itter, Anne Jung, Inga Claus, Sigrid Ahring, Tobias Warnecke, Rainer Dziewas, Paul Muhle

**Affiliations:** a Department of Neurology, University Hospital Muenster, Albert-Schweitzer-Campus 1, A1, 48149 Muenster, Germany; b Department of Neurology, Medical Faculty, University Hospital Düsseldorf, Moorenstraße 5, 40225 Düsseldorf, Germany; c Department of Neurology and Neurorehabilitation, Klinikum Osnabrueck, Am Finkenhügel 1, 49076 Osnabrueck, Germany

**Keywords:** Dysphagia, Pharyngeal electrical stimulation, Extubation failure, Stroke, Airway safety

## Abstract

Postextubation dysphagia is a major risk factor for extubation failure in acute stroke. Pharyngeal electrical stimulation (PES) is a novel neurostimulation technique for neurogenic dysphagia rehabilitation. We conducted a randomized controlled pilot trial evaluating PES early after extubation in acute stroke (N ​= ​60) focusing on dysphagia recovery trajectories and related outcomes until discharge. Patients with severe postextubation dysphagia, defined as Fiberoptic Endoscopic Dysphagia Severity Scale (FEDSS) score >4, received daily PES (real or sham, 10 ​min/day) for 3 consecutive days. By day 3, significantly fewer patients in the PES group exhibited persistent absence of spontaneous swallows (8 vs. 41 ​%) or pharyngeal sensory loss (4 vs. 55 ​%) compared to the sham group, indicating enhanced airway safety. Functional Oral Intake Scale (FOIS) score at day 3 was significantly higher in the PES group (4.1 vs 2.1 ​pts). FEDSS at days 5–7 and 8–10 showed a sustained treatment effect over time (2.4 vs. 3.7 ​pts. and 2.2 vs. 3.4 ​pts), resulting in better FOIS at discharge (4.7 vs. 3.5 pts.). PES shortened LOS in the intensive care unit (ICU) (3.1 vs. 8.5 days, p ​= ​0.008) and total hospital stay (13.8 vs. 21.9 days, p ​= ​0.004) from study inclusion. Tracheotomy rates were 13 vs. 33 ​% (p ​< ​0.067). The proportion of patients still cannulated at discharge (7 vs. 10 ​%) and the modified Rankin Scale at discharge (3.9 vs. 4.0) were comparable. PES enhanced recovery of postextubation dysphagia, improved airway safety and shortened length of ICU and hospital stay in acute stroke.

## Introduction

Endotracheal intubation and mechanical ventilation are frequently performed in severely affected acute stroke patients. While this treatment is often necessary and life-saving, timely extubation is desirable because prolonged mechanical ventilation is associated with higher pneumonia incidence, increased need for tracheostomy, longer length of stay in the intensive care unit (ICU), worse functional outcome, higher mortality, and increased health-care expenditures [1,2]. On the other hand, extubation failure is associated with the same adverse outcomes [[3], [4], [5]] and should be avoided.

A variety of different clinical parameters have been identified as predictors of extubation failure in mixed or neurological patient populations, in particular decreased level of consciousness [6], inability to follow commands [7], weak cough [8] and abundant bronchial secretions [2]. Recently, postextubation dysphagia (PED), as determined by flexible endoscopic evaluation of swallowing (FEES), has been proven as the most important risk factor for an unsafe airway and extubation failure in acute stroke patients [9]. PED may be exacerbated by dysphagia resulting from stroke itself, which occurs in a significant number of patients even without the need for intubation and deteriorates patient outcomes on multiple levels [10]. There is a clear correlation between dysphagia severity and the likelihood of reintubation, with patients showing aspiration of oropharyngeal secretions carrying the highest risk [9,11,12]. Although various treatments and management strategies exist to reduce complications from dysphagia after stroke [13], effective options that directly improve swallowing function in severely affected patients, particularly those with PED, remain limited. Unfortunately, there is no proven treatment for poststroke and especially postextubation dysphagia.

Pharyngeal electrical stimulation (PES) is an innovative approach to treating dysphagia by stimulating sensory pathways in the pharynx, which in turn triggers the release of substance P, a neuropeptide that enhances airway protective reflexes [14,15]. Research has demonstrated that PES helps facilitate corticobulbar projections and supports the reorganization of the cortical network involved in swallowing [16,17]. While studies on PES for dysphagia after stroke in a broad patient population have yielded mixed results [[18], [19], [20]], recent phase II and III trials have shown its effectiveness in improving swallowing function and aiding decannulation in severely dysphagic, tracheostomized stroke patients [[21], [22], [23]].

In the present randomized, sham-controlled clinical pilot trial, we evaluated the efficacy of PES early after extubation in acute stroke patients to treat severe postextubation dysphagia. Key primary outcomes from the trial have recently been reported in a short communication [24]: in brief, reintubation rate was 13 ​% following PES vs. 33 ​% after sham treatment (p ​= ​0.067). The pneumonia incidence was reduced (60 vs. 83 ​%, p ​= ​0.045). Greater overall improvement in swallowing function in the validated six-point Fiberoptic Endoscopic Dysphagia Severity Scale (FEDSS) [12], was observed in the short term (day 3) after PES (3.3 vs. 4.3 ​pts, p ​< ​0.0005). In the PES group, 73 ​% were on a totally oral diet upon discharge, compared to 47 ​% after sham intervention. The time to resume full oral feeding was 4.3 days in the PES group vs. 10.2 days in the sham group (p ​= ​0.001).

Here we present in more detail our study population and further results from an in-depth exploratory analysis of secondary outcomes related to swallowing function and airway safety throughout the clinical course until discharge. Insights will aid clinical decision-making for optimal patient selection and timing for PES application in the ICU setting.

## Participants and Methods

### Study outline and patients

This randomized controlled pilot trial (ClinicalTrials.gov NCT02470078) was conducted between December 2015 and April 2018 in our certified stroke unit with neurological ICU at the University Hospital Muenster. Consecutive adult acute stroke patients within 14 days of stroke onset who had been weaned from mechanical ventilation and had just been extubated were screened for study eligibility. Patients undergoing their first extubation attempt were included. The inclusion criterion was severe dysphagia as objectively assessed with FEES immediately postextubation and defined as FEDSS>4 (see “dysphagia assessment” below). The exclusion criteria were preexisting swallowing impairment unrelated to stroke, comorbidities potentially causing dysphagia, implanted electronic devices, or extubation for palliative care.

Participants were randomly assigned in a 1:1 ratio to receive either PES or sham stimulation using computer-assisted randomization. Randomization was performed within 4 ​h after extubation. Treatment started within 2 ​h thereafter. The randomization schedule was kept remotely from the study environment. The study coordinator provided assignment to the study physician who would deliver the intervention by phone.

Subjects were masked to their study group allocation (see “study intervention” below). The investigators who administered the treatment were not involved in any outcome assessment or other study-related activities. Investigators performing swallowing assessment, the ward physician and all other caregivers were masked. All patients continued to receive state-of-the-art medical and dysphagia care in accordance with national stroke and dysphagia guidelines throughout the study. This included daily visits by a speech and language therapist to perform medically indicated diagnostics and treatment tailored to the patient's inidvidual capabilities and needs (e.g. ice-chip-therapy, specific swallowing exercises depending on the individual dysphagia pattern, or even therapeutic feeding).

### Dysphagia assessment

FEES was performed as a gold standard in dysphagia diagnostics that is safe and well tolerated even in severely ill ICU patients [25]. Equipment consisted of a flexible rhinolaryngoscope (11101RP2, Karl Storz, Germany) with a light source and camera (rpCam-X, rpSzene®, Rehder/Partner, Germany). First, the parameters of airway safety, including ‘secretion management’, ‘spontaneous swallows’ and ‘laryngeal sensibility’, were evaluated following the “Standardized Endoscopic Swallowing Evaluation for Tracheostomy Decannulation” (SESETD) protocol [26,27] because of their known relevance for successful extubation. Each item was scored as “passed” (1 point) vs. “not passed” (0 points) with a sum score ranging from 0 to 3. ‘Saliva management’ was regarded as “not passed” if massive saliva pooling and/or silent penetration/aspiration of pooled saliva were permanently visible. The item ‘spontaneous swallows’ was considered failed if less than two swallows occurred within 2 ​min. Absent reaction after touching the arytenoids with the tip of the endoscope on both sides was scored as “fail“ for the item ‘laryngeal sensibility’.

Following airway safety evaluation, a validated stepwise FEES-protocol for dysphagia assessment in acute stroke [12] was applied, wich evaluates swallowing safety as well as efficacy, provides a specific diet recommendation for each score and is recommended by national guidelines [28]. Subjects were successively given standard volumes of puree consistency, liquids, and soft solid food. According to the risk of penetration or aspiration, or residue with saliva and different food consistencies, stroke-related dysphagia was classified based on the six-point FEDSS with 1 being the best and 6 being the worst outcome. Finally, the feeding status was rated using the Functional Oral Intake Scale (FOIS, ranging from 1 (nothing by mouth) to 7 (total oral diet with no restrictions)) [29].

### Study intervention: pharyngeal electrical stimulation (PES)

Stimulation was delivered via the Phagenyx catheter system and base station (Phagenesis Ltd, Manchester, UK). It consisted of a nasogastric feeding tube housing a pair of ring electrodes, which was connected to a stimulation device that delivered current intensities of 1–50 ​mA at 5 ​Hz. Catheter placement and stimulation were performed according to the manufacturer's instructions [22].

The treatment intensity (mA) was individually adjusted prior to every PES according to the perceptual threshold (PT) and maximum tolerated threshold (MTT) of the patient. The optimal stimulation intensity was automatically calculated by the device from the formula PT+0.75 ​× ​(MTT–PT). Severely disabled participants were asked to indicate PT and MTT with simple gestures, eye blinks, etc., depending on the patient's individual level of communication abilities, while the investigator who administered the treatment slowly increased the stimulation intensity. Stimulation intensities (PT, MTT and calculated optimal stimulation intensity) were recorded for each PES intervention session.

In the sham group, the optimization procedure was performed as similar as possible to active PES. Afterward, the catheter was left in place but no current was applied. The intervention was delivered on three consecutive days for 10 ​min daily.

### Endpoints and parameters

To characterize our patient cohort, information on patient demographics, stroke characteristics and acute stroke treatment was collected. We also kept records on the reason for initial intubation, time from admission to study inclusion and duration of ventilation. The National Institute of Health Stroke Scale (NIH-SS) score was noted upon admission and prior to the first stimulation. The Glasgow Coma Score (GCS) and Richmond Agitation Sedation Scale (RASS) score were documented at the time of study inclusion.

Parameters of airway safety and swallowing function according to the SESETD protocol and the FOIS were assessed at day 3. As a follow-up, FEDSS was reassessed on days 5–7 and 8–10 in those participants who had not been discharged or transferred to another hospital. Need for a tracheostomy after reintubation and presence of a tracheal cannula at discharge were documented. The length of stay (LOS) in the ICU and overall hospital LOS, both measured from study inclusion were analyzed. Moreover, the final FOIS and global functional outcome (modified Rankin Scale, mRS) including cases of death were recorded at discharge.

### Sample size calculation

The sample size calculation for this pilot study was based on the available evidence when the study was designed. It was powered for the primary outcome parameter “need for reintubation”, that was previously published [24]. A meta-analysis reported extubation failure rates of up to 40 ​% in neurological ICU populations [6]. The first randomized trial on PES in tracheotomized stroke patients revealed an almost four-times higher decannulation success rate compared to sham stimulation [21]. Similarly, we expected a fourfold reduction in the need for reintubation, i.e., from 40 to 10 ​%. A sample size of 30 patients per group yielded a power of 80 ​% for the detection of a difference at a one-sided 0.05 significance level for a reduction in the need for reintubation by approximately 30 ​% with PES compared to sham treatment. Patient enrollment was stopped once the target sample size was reached.

### Statistical analysis

All patients were included in an intention-to-treat analysis. Comparison of the PES and sham treatment groups was performed using the *t*-test for parametric continuous variables, the Mann‒Whitney *U* test for nonparametric continuous and ordinal variables, and the chi-square or Fisher exact test for categorical parameters. The log-rank (Mantel‒Cox) test was used to compare Kaplan‒Meier curves for lengths of stay. Cox Proportional-Hazards regression was used to calculate Hazard Ratios for being discharged. One-way analysis of variance for repeated measures assessed changes in PES treatment intensities over time. A p-value of <0.05 indicated statistical significance. Data analysis was carried out using SPSS 27.0 (IBM Corp., USA).

## Results

**Fig. 1:**
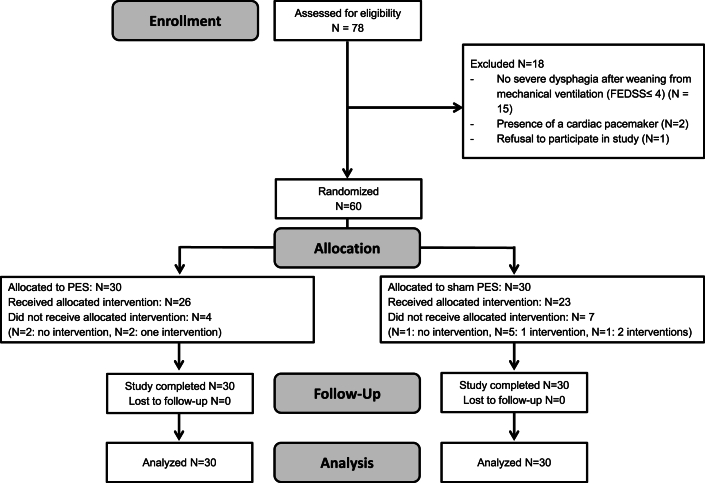
Patient recruitment flowchart (FEDSS = Fiberoptic Endoscopic Dysphagia Severity Score, PES ​= ​pharyngeal electrical stimulation).

Sixty patients were enrolled and randomized, all of whom could well tolerate catheter placement and stimulation. As reported previously [24], not all patients received the allocated intervention as per protocol, mainly due to need for reintubation during the three-day intervention or before post-intervention FEES at day 3 could be performed. Fig. 1 shows the details of patient recruitment. Nobody was lost to follow-up.

Table 1 shows the baseline characteristics of the study participants. Study groups only differed in NIH-SS upon admission prior to acute stroke treatment, but no longer at the time of study inclusion. Baseline parameters of airway safety and swallowing function including FEDSS, FOIS and SESEDT were comparable.

**Table 1:** Baseline data of the stimulation and control groups.

	Treatment Group (N ​= ​30)	Control Group (N ​= ​30)	p value
Age (years)	69.5 ​± ​15.7	64.6 ​± ​12.7	0.185
Female, n (%)	19 (55)	13 (43)	0.121
NIH-SS (pts, upon admission)	16.4 ​± ​3.6	14.3 ​± ​4.1	0.041^a^
NIH-SS (pts, upon study inclusion)	13.7 ​± ​2.9	12.5 ​± ​3.0	0.09
GCS (pts, upon study inclusion)	13.9 ​± ​1.0	14.3 ​± ​0.8	0.140
RASS (pts, upon study inclusion)	−0,2 ​± ​0.4	−0.2 ​± ​0.4	0.710
**Type of stroke, n (%)**			
Ischemic stroke	27 (90)	29 (97)	0.301
Hemorrhagic stroke	3 (10)	1 (3)	
**Site of stroke, n (%)**			
Infratentorial	6 (20)	2 (7)	0.129
Supratentorial	24 (80)	28 (93)	
Right/Left supratentorial, n	13/11	20/8	0.198
**Stroke etiology, n (%)**			
Large-artery atherosclerosis	9 (33)	5 (17)	0.165
Cardioembolism	15 (56)	16 (55)	0.977
Small-vessel occlusion	0 (0)	0 (0)	n.a.
Other determined etiology	1 (4)	4 (14)	0.186
Unknown etiology	2 (7)	4 (13)	0.440
**Vascular risk factors, n (%)**			
Arterial hypertenson	27 (90)	24 (80)	0.278
Hyperlipidemia	20 (67)	19 (63)	0.787
Diabetes mellitus	7 (23)	9 (30)	0.559
Smoking	5 (17)	9 (30)	0.222
**Acute stroke treatment, n (%)**			
i.V. Thrombolysis	16 (59)	11 (38)	0.110
Mechanical recanalization	24 (89)	26 (90)	0.926
Decompressive craniectomy/hematoma evacuation	5 (17)	4 (13)	0.718
**Reason for intubation, n (%)**			
Acute respiratory distress	0 (0)	0 (0)	n.a.
Decreased protective reflexes	3 (10)	4 (13)	0.688
Planned intervention/surgery	27 (90)	26 (87)	
Duration of mechanical ventilation (h)	83.3 ​± ​95.3	64.7 ​± ​61.3	0.264
Time from admission to study inclusion (d)	3.3 ​± ​2.5	2.5 ​± ​2.6	0.066
**Swallowing function at baseline**			
FEDSS	5.4 ​± ​0.5	5.3 ​± ​0.5	0.288
Saliva pooling	14 (47)	11 (37)	0.432
No spontaneous swallowing	14 (47)	16 (53)	0.606
Sensory loss	22 (73)	20 (67)	0.573
SESEDT- sum score	1.3 ​± ​1.1	1.4 ​± ​1.0	0.499
FOIS	1.0 ​± ​0.0	1.0 ​± ​0.0	1.000

*Data represent the mean ​± ​standard deviation unless otherwise stated. (FEDSS* ​= ​*Fiberoptic Endoscopic Dysphagia Severity Scale, FOIS* ​= ​*Functional Oral Intake Scale, GCS ​= ​Glasgow Coma Scale; NIH-SS* ​= ​*National Institute of Health Stroke Scale; RASS* ​= ​*Richmond Agitation-Sedation Scale, SESEDT* ​= ​*Standardized Endoscopic Swallowing Evaluation for Tracheostomy Decannulation.*

a Indicates statistical significance*)*.

### Treatment effects

**Fig. 2:**
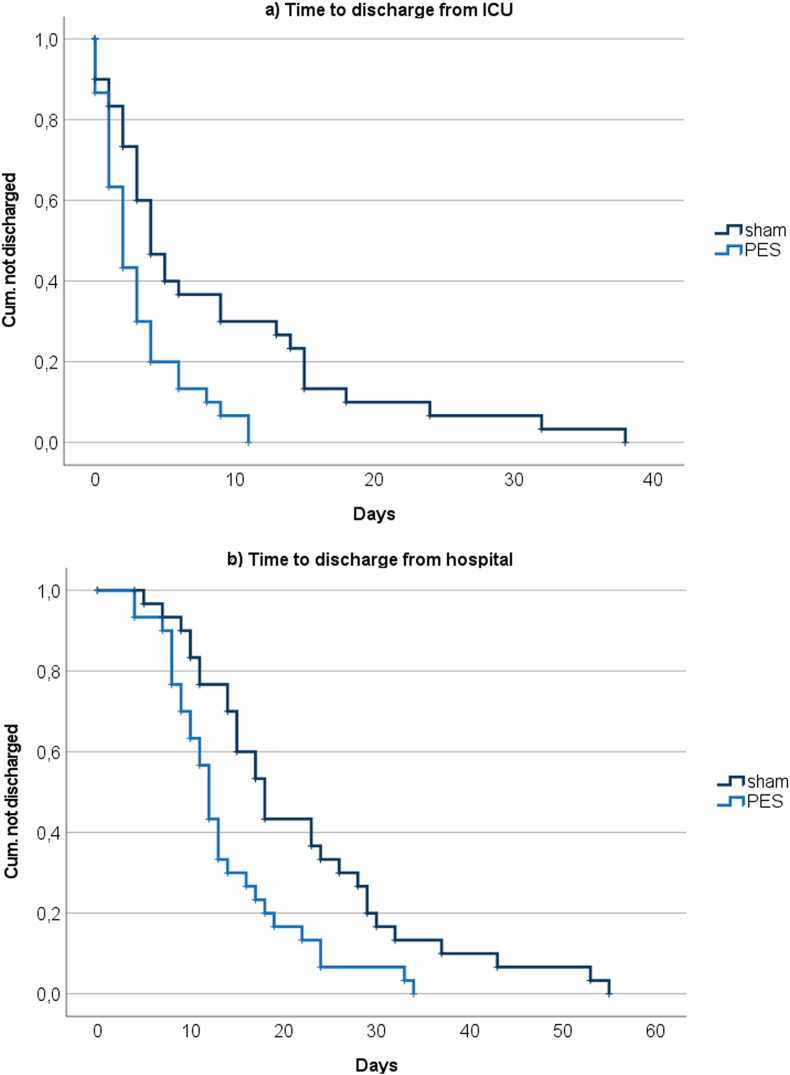
Kaplan‒Meier curves of the PES and sham treatment groups for (**a**) length of stay in the ICU (p ​= ​0.001; log-rank Mantel‒Cox, Hazard Ratio (HR): 2.3), and (**b**) length of stay in the hospital (p ​= ​0.003; log-rank Mantel‒Cox, HR: 2.1), both from study inclusion (ICU ​= ​intensive care unit, PES ​= ​pharyngeal electrical stimulation).

Intervention effects are listed in Table 2. After three days of PES, patients showed significantly more spontaneous swallows, better pharyngeal sensory function and a higher SESETD score, indicating better swallowing function and more efficient airway management. Accordingly, the FOIS score on day 3 was significantly better after PES. Endoscopic follow–up investigations of the FEDSS at days 5–7 and 8–10 were only possible in a subgroup of patients who were not yet transferred to rehabilitation centers. Nonetheless, the findings in this subgroup analysis showed that the positive treatment effect of PES on swallowing function sustained over time (2.4 vs. 3.7 FEDSS points and 2.2 vs. 3.4 FEDSS points, respectively). The PES group also showed a higher FOIS score at discharge than the sham group. ICU stay (3.1 vs. 8.5 days, p ​= ​0.008) and acute-care hospital stay (13.8 vs. 21.9 days, p ​= ​0.004) from the time of study inclusion were both significantly shortened by PES (for details see Kaplan‒Meier curves Fig. 2a and b). According to our local routine, all reintubated patients were tracheostomized without further extubation attempts. The proportion of patients still having a tracheal cannula at discharge was similar between groups. The modified Rankin Scale at discharge was comparable. There were no cases of death.

**Table 2:** Intervention effects.

	Treatment Group	Control Group	Odds ratio or median difference (95 ​% CI)	p value
SESEDT, day 3	2.8 ​± ​0.5	2.0 ​± ​0.8	−1 (−1–0)	<0.0005^a^
Saliva pooling	1 (4)	1 (5)	0.84 (0.049–14.261)	1.000
No spontaneous swallowing	2 (8)	9 (41)	0.120 (0.023–0.642)	0.006
Sensory loss	1 (4)	12 (55)	0.033 (0.004–0.291)	<0.0005^a^
FOIS, day 3	4.1 ​± ​1.6	2.1 ​± ​1.6	−2 (−3–−1)	<0.0005^a^
FEDSS, day 5–7	2.4 ​± ​1.0 (N ​= ​23)	3.7 ​± ​0.9 (N ​= ​19)	1 (1–2)	<0.0005^a^
FEDSS, day 8–10	2.2 ​± ​1.2 (N ​= ​12)	3.4 ​± ​1.2 (N ​= ​14)	−1 (−4–0)	0.020^a^
FOIS at discharge	4.7 ​± ​2.0 (N ​= ​30)	3.5 ​± ​2.1 (N ​= ​30)	−1 (−3–0)	0.017^a^
LOS in the ICU from study inclusion (d)	3.1 ​± ​3.1	8.5 ​± ​9.5	2 (1–5)	0.008^a^
LOS in hospital from study inclusion (d)	13.8 ​± ​7.4	21.9 ​± ​12.6	6 (2–11)	0.004^a^
Tracheotomy after reintubation, n (%)	4 (13)	10 (33)	0.308 (0.084–1.127)	0.067
Tracheal cannula at discharge, n (%)	2 (7)	3 (10)	0.235 (0.044–1.241)	0.145
mRS at discharge, (pts)	3.9 ​± ​0.9	4.0 ​± ​1.0	0 (0–0)	0.674
Death, n (%)	0 (0)	0 (0)	n.a.	n.a.

*(N (%), FEDSS* ​= ​*Fiberoptic Endoscopic Dysphagia Severity Score, FOIS* ​= ​*Functional Oral Intake Scale; ICU ​= ​intensive care unit; IMC ​= ​intermediate care unit; LOS ​= ​length of stay; mRS ​= ​modified Rankin Scale; SESEDT* ​= ​Standardized Endoscopic Swallowing Evaluation for Tracheostomy Decannulation.

a Indicates statistical significance*).*

### Indicators of treatment response in the PES group

The small number of reintubated and tracheotomized patients in the PES group (N ​= ​4) precluded a robust analysis of treatment success indicators. Regarding PES stimulation parameters in successfully simulated patients, i.e. not needing reintubation and tracheotomy, one-way analysis of variance for repeated measures revealed a significant decrease in perceptual threshold from session to session ((mA) 1st PES: 18.3 ​± ​8.0, 2nd: 16.2 ​± ​7.8, 3rd: 13.2 ​± ​6.3; F(2, 50) ​= ​10.427, p ​< ​0.001) and maximum tolerated threshold ((mA) 1st PES: 41.5 ​± ​8.4, 2nd: 36.0 ​± ​11.1, 3rd: 31.7 ​± ​11.1; F(2, 50) ​= ​12.807, p ​< ​0.001). The same applies to the calculated optimal stimulation intensity ((mA) 1st PES: 35.5 ​± ​7.6; 2nd: 31.1 ​± ​9.9, 3rd: 27.2 ​± ​9.3; F(2, 50) ​= ​14.101, p ​< ​0.001).

## Discussion

This is the first randomized controlled trial assessing the effect of early PES on postextubation dysphagia in ICU-related stroke patients aiming to prevent dysphagia-related complications. The intervention resulted in a lasting and significantly greater improvement in severe dysphagia among recently extubated stroke patients compared to the sham treatment, leading to enhanced airway safety and reduced length of stay in both the ICU and acute care hospital. As recently reported [24], the primary endpoint, “need for reintubation”, scarcely missed significance in this small pilot study. Key secondary endpoints achieved statistical significance, demonstrating that PES substantially lowered the risk of pneumonia and reduced use of antibiotics [24].

PES-induced faster recovery of swallowing function and airway safety, as documented here, may contribute to a reduction in a patient's overall intensive care treatment risk because of the reduced need for invasive procedures such as PEG tube placement and tracheostomy, which are generally safe measures but nevertheless associated with their own potential complications [30,31]. Stroke-associated pneumonia as a consequence of severe dysphagia is independently associated with poor functional outcome [32] and increased mortality [33]. Any measures to prevent these complications – such as timely PES - should be undertaken. Further, a recent study has demonstrated that particularly severe dysphagia with impaired secretion management is associated with additional health-care costs [34]. Conversely, a shorter length of stay, lower incidence of pneumonia and less use of antibiotics can substantially reduce the incremental costs of care related to complications in acute stroke [35]. Last but not least, better recovery of swallowing function with an earlier return to an oral diet means a substantial increase in a patient's quality of life and reduces dependency on institutional care.

### Timing of PES treatment

PES was initiated within 6 ​h of extubation, consistent with current evidence from other PES trials indicating that treatment initiation at the earliest convenience is beneficial [[21], [22], [23],^36^,^37^]. Thinking ahead, it may be more effective to treat orally intubated stroke patients at high risk for severe PED and associated airway complications even before an extubation trial. This requires identifying patients likely to suffer from PED and who may benefit from such early PES while still intubated. The previously proposed “Determine Extubation Failure in Severe Stroke” (DEFISS) score [9] offers a feasible tool for risk stratification of dysphagia severity and reintubation need in this situation. It includes clinical assessment of oral motor function and easy-to-collect clinical data such as duration of ventilation, stroke lesion location and stroke severity. Pilot data using DEFISS indicated that PES prior to extubation may reduce extubation failure risk and shorten time to discharge in orally intubated and mechanically ventilated stroke patients at high risk of severe dysphagia [38].

### PES stimulation intensity and related pathophysiological considerations

The primary working principle of PES is neuromodulation, i.e., enhancing excitability and neuroplasticity in the swallowing sensorimotor network of the brain by stimulating afferent sensory pathways [17,39]. Intact sensory function is crucial for the motor output of swallowing. Under experimental conditions, pharyngeal anesthesia reduces activation in the cortical sensorimotor swallowing network [40], thereby deteriorating swallowing performance even in healthy subjects [41]. Clinically, stroke lesions of the primary sensory cortex are especially predictive of severe swallowing dysfunction [42]. Dysphagia severity in stroke has been associated with the degree of pharyngeal sensory impairment [43,44]. In addition to damage to the central swallowing network itself, more peripheral mechanisms, including sensory impairment due to pharyngolaryngeal lesions caused by the tube and critical illness neuropathy and myopathy which leads to muscle weakness and dyscoordination of breathing and swallowing of ICU-related dysphagia, apply to acute stroke patients. Taken together, impaired sensory function is a key pattern of ICU-acquired dysphagia [45] that is specifically targeted by PES, both centrally and peripherally: By applying a peripheral virtual sensory lesion model to healthy subjects mimicking the situation in ICU patients, we previously demonstrated that PES is also able to revert the detrimental effects of such peripheral sensory impairment on swallowing processing [46]. The local mechanism of action is most likely a PES-induced release of Substance P from pharyngeal sensory nerve endings [14]. Substance P is a neuropeptide known to enhance the swallow reflex. An increase after PES has been related to decannulation success in severely dysphagic tracheostomized stroke patients [15].

Stimulation intensity in the current study (35.5 ​mA on average for the first intervention) was similar to that reported in a recent multicenter RCT on tracheostomized stroke patients with severe dysphagia [22]. PES treatment responders showed a significant decrease in PTT and MTT over time as an indicator of sensory recovery. This is also a finding consistent with an earlier study in tracheostomized stroke patients [21]. In the non-stroke neuro-ICU population, a lower PTT at baseline as a surrogate marker for relatively preserved pharyngeal sensory function was found to be a predictor for a quicker improvement of PED after PES [37]. Better pharyngeal sensation not only improves swallowing function in terms of nutrition but also contributes to more effective management of secretions and fewer airway complications. The additional peripheral mechanism of action of PES makes it a potentially valuable treatment option for nonstroke/nonneurological ICU patients, in whom dysphagia due to the aforementioned reasons is increasingly being acknowledged as a relevant cause for delayed extubation or decannulation and is associated with poor outcomes in survivors of critical illness [[47], [48], [49]].

### Limitations

This was a pilot single-center RCT with a small sample size, and the results need to be re-examined by a larger multicenter study. Our study population was restricted to stroke patients. The results should be generalized with caution to other neurological conditions or the broader ICU population as the underlying causes for PED may vary. From a pathophysiological point of view, however, there is justified hope that PES can also improve PED related to purely peripheral sensory dysfunction that can occur with any critical illness or as a consequence of ICU treatment. In accordance with this hypothesis, in the previously published large-scale multi-centre observational cohort study, the PHAryngeal electrical stimulation for treatment of neurogenic Dysphagia European Registry (PHADER), PES significantly improved diet advancement by reducing dysphagia severity and aspiration risk in patients with neurogenic dysphagia of various origin [23].

In conclusion, timely PES enhances recovery of postextubation dysphagia, improves airway safety and shortens length of ICU stay in acute stroke. Future trials should confirm these results in neurological and nonneurological ICU populations and further explore the effect of timely PES, i.e. prior to an extubation trial, in intubated patients at high risk of severe dysphagia.

## Ethics approval and consent to participate

The study was performed in line with the principles of the Declaration of Helsinki. Approval was granted by the local ethics committee at the University of Muenster. Informed consent was obtained from all patients or their legal representative in case the patient's communication was impaired. Trial registration: ClinicalTrials.gov NCT02470078 (June 12, 2015).

## Consent for publication

Not applicable.

## Availability of data and materials

The datasets analyzed during the current study are available from the corresponding author upon reasonable request.

## Author Contributions

SSK, RD and PM had full access to all of the data in the study and take responsibility for the integrity of the data and the accuracy of the data analysis. SSK, RD, TW and PM designed the study; SSK, RD, PM, BL, IC and SA were involved in data acquisition; SSK, RD, PM, JvI and AJ performed the analysis and interpretation of the data. SSK and RD drafted the article, and all authors provided critical revision of the article and gave final approval of the version submitted for publication.

## Funding

This work was supported by the German Research Foundation (SU 922/1-1, DZ 78/1-1). SSK is supported by the Else Kröner-Fresenius-Stiftung with an endowed professorship. BL was supported by a clinician scientist grant from the medical faculty of the University of Muenster.

## Declaration of competing interest

The authors declare the following financial interests/personal relationships which may be considered as potential competing interests: Sonja Suntrup-Krueger reports financial support was provided by German Research Foundation. Sonja Suntrup-Krueger reports financial support was provided by Else Kroner-Fresenius Foundation. Rainer Dziewas reports a relationship with Phagenesis Ltd that includes: board membership and speaking and lecture fees. Sonja Suntrup-Krueger reports a relationship with Phagenesis Ltd that includes: speaking and lecture fees. Paul Muhle reports a relationship with Phagenesis Ltd that includes: speaking and lecture fees. Bendix Labeit reports a relationship with Phagenesis Ltd that includes: speaking and lecture fees. If there are other authors, they declare that they have no known competing financial interests or personal relationships that could have appeared to influence the work reported in this paper.
